# Calcitonin gene-related peptide causes migraine aura

**DOI:** 10.1186/s10194-023-01656-4

**Published:** 2023-09-07

**Authors:** Haidar M. Al-Khazali, Håkan Ashina, Astrid Wiggers, Kathrine Rose, Afrim Iljazi, Rune Häckert Christensen, Henrik Winther Schytz, Faisal Mohammad Amin, Messoud Ashina

**Affiliations:** 1 Department of Neurology, Danish Headache Center, Copenhagen University Hospital – Rigshospitalet, Copenhagen, Denmark; 2 Department of Clinical Medicine, Faculty of Health and Medical Sciences, University of Copenhagen, Copenhagen, Denmark; 3 Department of Anesthesia, Critical Care and Pain Medicine, Beth Israel Deaconess Medical Center, Boston, MA USA; 4 Harvard Medical School, Boston, MA USA; 5 Department of Brain and Spinal Cord Injury, Copenhagen University Hospital – Rigshospitalet, Copenhagen, Denmark

**Keywords:** Trigeminovascular System, Cortical spreading depolarization, Headache disorders, Pathophysiology

## Abstract

**Background:**

Although the involvement of calcitonin gene-related peptide (CGRP) in migraines is well-established, its specific role in investigating the aura phase, which often precedes the headache, remains largely unexplored. This study aims to instigate CGRP’s potential in triggering aura, thus establishing its role in the early stages of migraine.

**Methods:**

In this open-label, non-randomized, single-arm trial, 34 participants with migraine with aura received continuous intravenous infusion of CGRP (1.5 µg/min) over 20 min on a single experimental day. Participants were required to be free of headache and report no use of acute medications 24 h before infusion start. The primary endpoint was the incidence of migraine aura during the 12-hour observational period after the start of infusion.

**Results:**

Thirteen (38%) of 34 participants developed migraine aura after CGRP infusion. In addition, 24 (71%) of 34 participants developed migraine headache following CGRP infusion.

**Conclusions:**

Our findings suggest that CGRP could play an important role in the early phases of a migraine attack, including during the aura phase. These insights offer a new perspective on the pathogenesis of migraines with aura. They underscore the need for additional research to further explore the role of CGRP in these initial stages of a migraine attack, and potentially inform future development of therapeutic interventions.

**Trial registration:**

ClinicalTrials.gov Identifier: NCT04592952.

**Graphical Abstract:**

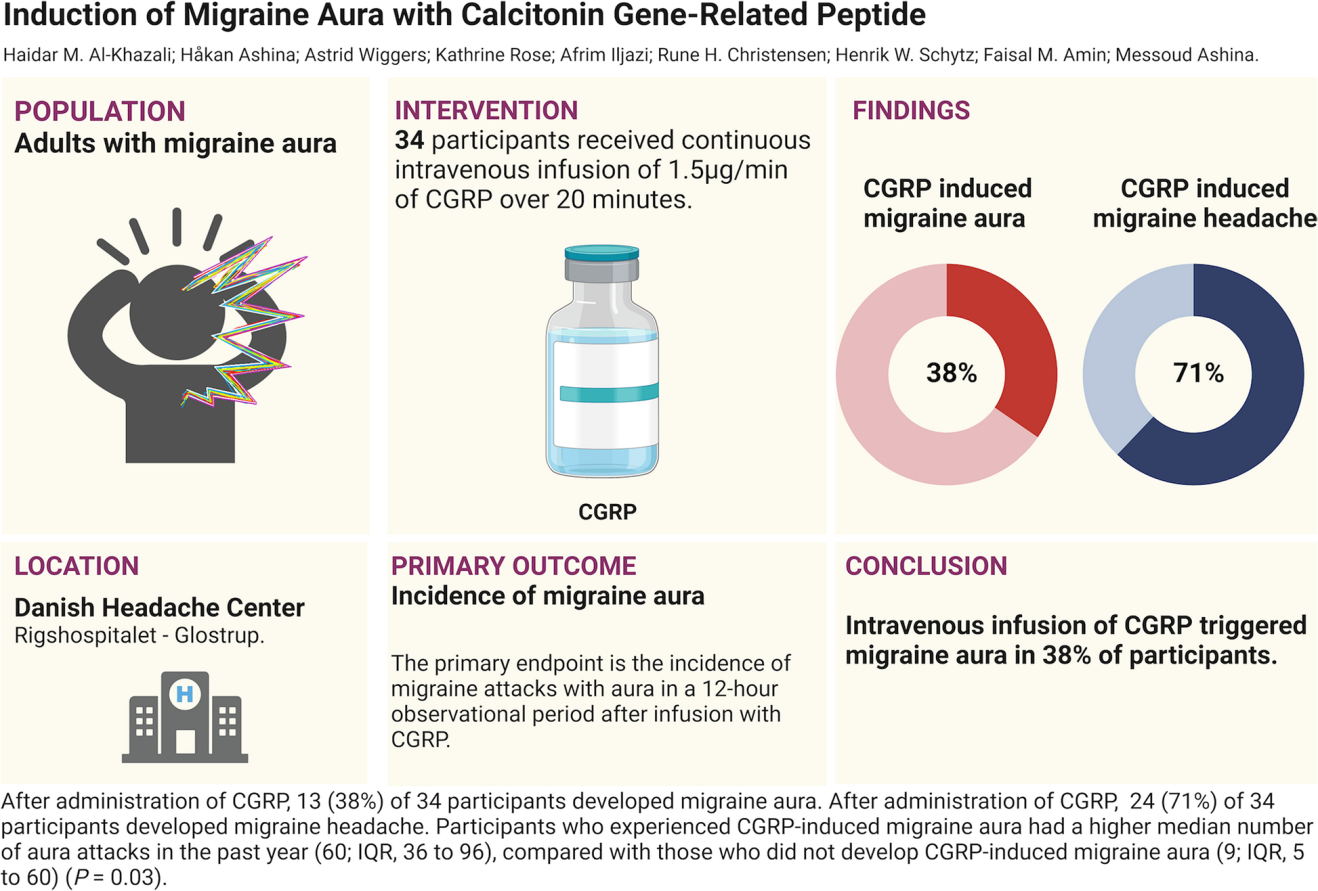

**Supplementary Information:**

The online version contains supplementary material available at 10.1186/s10194-023-01656-4.

## Background

Migraine is a prevalent and disabling neurological disorder that affects over 1 billion people worldwide [1]. The clinical presentation is characterized by recurrent attacks of headache accompanied by symptoms such as photophobia, phonophobia, and nausea/vomiting [2]. About 30% of people with migraine also experience aura which is defined as transient, focal neurological symptoms that precede or accompany the headache phase of a migraine attack [3]. The most common aura symptoms include visual disturbances (e.g., as a fortification spectra) and predominantly unilateral paraesthesia (e.g., numbness) that gradually spreads on the face or arm [2, 3].

The neurobiological substrate of aura is believed to be cortical spreading depolarization (CSD), a self-propagating wave of neuronal and glial excitation across the cortex followed by inhibition [4]. The prevailing theory posits that CSD triggers the release of pronociceptive mediators, leading to the dilation of meningeal arteries and activation of perivascular primary afferents, thereby causing headache [4, 5].

However, this theory is largely derived from clinical observations that aura often precedes the onset of headache, and it must be considered with caution due to significant interspecies variations between human and animal CSD. Recent publications have further highlighted the complexities and potential problems with translating findings from animal models to human migraine pathology [6]. As such, while the link between CSD and headache has been long discussed, it remains an area of ongoing investigation, and the question of whether shared disease mechanisms underlie both the aura and headache phase of a migraine attack continues to be an open and complex question [5]. In this context, signalling molecule calcitonin gene-related peptide (CGRP) has received increased attention due to the effectiveness of drugs targeting CGRP signalling in treating migraine, as well as self-reported aura [7–9]. Furthermore, 1 experimental study found that CGRP might be able to induce aura in some people with migraine with aura [10]. The study was however limited by a small sample, precluding us from drawing firm conclusions about the involvement of CGRP in the genesis of migraine attacks with aura [10]. Moreover, it is unknown whether the induction of aura symptoms depends on the frequency of aura. If so, it might be speculated that a higher frequency of aura increases the susceptibility to develop CGRP-induced migraine attacks with aura.

In the present open-label, non-randomized, single-arm trial, adult people with migraine with aura were enrolled and allocated to receive a 20-minute continuous intravenous infusion of CGRP on a single experimental day. The aim was to investigate whether CGRP infusion can induce migraine attacks with aura in people with migraine with aura.

## Methods

### Study Oversight

 Registry for Migraine (REFORM) is a prospective, single-centre study of adult people with migraine that was conducted at the Danish Headache Center [11]. The present data was collected as part of the larger parental REFORM study. The study protocol was approved by the relevant ethics committee, and all participants provided written informed consent before the commencement of any study-related assessments or procedures. The study was conducted in accordance with the principles of the Declaration of Helsinki. The study was also registered at ClinicalTrials.gov (NCT04592952).

### Study Population

The study enrolled adult participants diagnosed with migraine with aura based on the criteria outlined in the International Classification of Headache Disorders, 3rd edition (ICHD-3) [12]. In addition, participants needed to have an average of ≥ 4 monthly migraine days in the 3 months preceding enrolment and ≥ 1 day with migraine with aura in the past year. Participants were also required to report onset of migraine before the age of 50 years and have a documented history of migraine for a minimum of 12 months prior to enrolment. Eligibility also extended to participants who were already receiving stable doses of preventive medication for migraine. The exclusion criteria were primarily a history of headache disorders other than migraine as well as pregnancy or breastfeeding. For a complete list of inclusion and exclusion criteria, the readers are referred to the Supplemental Appendix Table 1.

### Study Design and Procedures

This open-label, non-randomized, single-arm trial was conducted at the research facilities of a tertiary care unit from October 2020 to January 2022. The study consisted of 1 screening visit and 1 experimental day. Prior to the experiment, participants had no restrictions on consuming caffeine, cacao, or alcohol as usual. However, they needed to confirm that they hadn’t used any headache medications or experienced a headache within 24 h before the infusion started. During the screening visit, participants underwent a semi-structured interview and a physical examination performed by trained personnel. An electrocardiogram was then performed, and the participants were instructed that CGRP might induce headache. No specific details were disclosed on the possible onset and characteristics of the headache. All participants received a 20-minute continuous intravenous infusion of αCGRP (1.5 µg/min), and the administered dose and timing was identical to the one used in previous human experimental studies [13, 14]. Drug preparation was performed by independent hospital pharmacy staff.

The participants were placed in a supine position, and the antecubital fossa was cannulated to gain intravenous access. A time- and volume-controlled infusion pump was used to administer CGRP, and vital signs (i.e., blood pressure and heart rate) were recorded throughout the 1-hour in-hospital phase. At the time of infusion start, a headache diary was used to collect outcome data on clinical characteristics (including aura), adverse events, and use of rescue medication. These items were then recorded every 10 min until 1 h after the infusion started. Participants were then discharged and asked to complete headache diary hourly until 12 h post-infusion start. If aura symptoms occurred, participants were instructed to fill out a diagnostic aura diary and perform drawings of the visual and sensory aura symptoms. Moreover, participants were allowed, if needed, to treat their headache with rescue medication (e.g., sumatriptan).

### Endpoints

The primary endpoint of the study was the incidence of migraine aura within the 12-hour observational period following the initiation of CGRP infusion. Migraine aura was defined based on the fulfilment of criteria B and C for migraine with aura according to ICHD-3 [12] (see the Supplemental Appendix Table 2A). The secondary endpoint was the incidence of migraine headache within the same 12-hour observational period after CGRP infusion. Migraine headache was defined as meeting criteria C and D for migraine without aura according to ICHD-3 [12] (see the Supplemental Appendix Table 2B), or as the participant reporting a headache that resembles their typical migraine headache and necessitates the use of rescue medication during the 12-hour observational period.

### Statistical analysis

Normally distributed data are presented as mean values ± standard deviation or range, while non-normally distributed data are reported as median values with interquartile range (IQR) or range. The normality of the data was assessed using the Shapiro-Wilk’s test. The baseline was defined as the time of infusion start. Categorical data are presented as counts and proportions. The Wilcoxon rank sum test was used to compare the median number of yearly aura attacks between participants who developed migraine aura and those who did not. Significance was accepted at *P* < 0.05. All statistics were performed in R version 4.1.0.

## Results

### Characteristics of the study population

Thirty-four participants with migraine with aura completed the study. The mean age was 43 ± 14 years, and most participants were women (*n* = 30). The mean body mass index was 23 ± 4 kg/m^2^, and 29 (85%) of 34 participants had a diagnosis of migraine with and without aura, whilst 5 (15%) had a diagnosis of migraine with aura exclusively. The median number of migraine attacks with aura was 25 (IQR, 7 to 72) in the past year, and 17 (81%) participants reported current use of preventive medication for migraine. Regarding the aura type, 18 (53%) participants experienced exclusively visual aura, 14 (41%) had both visual and sensory aura, and 2 (6%) reported exclusively sensory aura. Table 1 summarizes the characteristics of the study population.

**Table 1:** Study population characteristics

	CGRP induced migraine aura (*n* = 13)	CGRP induced migraine without aura (*n* = 11)	CGRP did not induce migraine with or without aura (*n* = 10)
Age, mean (SD)	43.2 (11.6)	39.3 (16.8)	42.4 (11.8)
Women/ men, *n* (%)	12/1 (92% / 8%)	11 (100%)	7 / 3 (70% / 30%)
Episodic migraine, *n* (%)	9 (70%)	5 (45%)	5 (50%)
Chronic migraine, *n* (%)	4 (30%)	6 (55%)	5 (50%)
Migraine with and without Aura, *n* (%)	9 (70%)	10 (90%)	10 (100%)
Migraine with aura exclusively, *n* (%)	4 (30%)	1 (9%)	0 (0%)
Number of monthly headache days, median (range)	14 (4–20)	10 (4–17)	13.5 (4–20)
Number of monthly migraine days, median (range)	8 (4–20)	7 (4–15)	9 (4–20)
Frequency of aura last year, median (range)	60 (24–179)	8.6 (1-120)	8 (1–72)
Visual aura, *n* (%)	12 (92%)	11 (100%)	10 (100%)
Sensory aura, *n* (%)	11 (85%)	4 (37%)	2 (20%)
Current preventive medication, *n* (%)	11 (85%)	4 (37%)	6 (60%)

### Migraine aura

During the 12-hour observational period following the start of CGRP infusion, 13 (38%) of 34 participants experienced migraine aura (Table 2). The CGRP-induced aura symptoms predominantly consisted of visual disturbances (*n* = 10), while sensory disturbances were less common (*n* = 7). Of the 13 participants, 11 had the onset of migraine aura occur during the 60-minute in-hospital phase.

**Table 2:** Clinical characteristics of aura after calcitonin gene-related peptide provocation

Participant no.		Onset of aura (min)	Duration of aura (min)	Gradually spreading	Localization of aura	Description	Headache onset (min)	Localization of headache	Mimics usual aura	Fulfil aura criteria	Time between aura onset and headache onset
1	Spontaneous	~	Visual, 40 Sensory, 45 min	Yes	Right side	Visual: Flickering, foggy	~	Right or left side: Parietal, frontal, periorbital.	~	Yes	-60
	CGRP infusion	360	Visual, 60	Yes	Right side	Visual: Flickering, foggy	40	Rigth side: Parietal, temporal, Occipital. Left side: Frontal, Parietal, temporal, occipital.	Yes	Yes	320
2	Spontaneous	~	Visual, 8 Sensory, 5	Yes	Left side	Visual: Scotoma, flicking, zigzag lines Sensory: numbness	~	Left side: Temporal, frontal, periorbital.	~	Yes	-180
	CGRP infusion	120	Sensory, 15 min	Yes	Left side fingers and hand	Sensory: Tingling, numbness	10	Rigth side: Parietal, temporal, occipital. Left side: Frontal, parietal, temporal, occipital.	Yes	Yes	110
3	Spontaneous	~	Visual, 30 Sensory, 30	Visual: No Sensory: Yes	Visual: Left or right side Sensory: Left side	Visual: Scotoma, zigzag lines Sensory: Tingling	~	Left side: Frontal, parietal, temporal, occipital, periorbital.	~	Yes	-30
	CGRP infusion	20	Visual, 20 Sensory, 40	Visual: Yes Sensory: Yes	Visual: Left side Sensory: Left side	Visual: Flickering, zigzag lines Sensory: Tingling in fingers, arm, face and tung	10	Right side: Frontal, temporal, occipital. Left side: Frontal, parietal, temporal, occipital, periorbital.	Yes	Yes	10
4	Spontaneous	~	Visual, 60 Sensory, 60	Yes	Visual: Left or right side Sensory: Left or right side	Visual: Scotoma, black dots Sensory: numbness	~	Right side: Frontal, occipital. Left side: Frontal, temporal, occipital.	~	Yes	-60
	CGRP infusion	10	Visual, 20	Yes	Left side	Visual: Flicking, black dots	10	Right side: Frontal Left side: Frontal, temporal.	Yes	Yes	0
5	Spontaneous	~	Visual, 30 Sensory, 720	Yes	Visual: Left side Sensory: Left side	Visual: Flicking, white dots Sensory: Numbness	~	Left or right side: Temporal, occipital.	~	Yes	-60
	CGRP infusion	30	Visual, 30	Yes	Left side	Visual: Central white dots	10	Right side: Frontal. Left side: Frontal, temporal.	Yes	Yes	20
6	Spontaneous	~	Sensory, 30	Yes	Left side	Sensory: Numbness	~	Left side: Periorbital and occipital.	~	Yes	-30
	CGRP infusion	30	Sensory, 10	Yes	Sensory: Left side, face, hand	Sensory: Numbness	10	Right side: Frontal, parietal, temporal, Occipital. Lift side: Frontal, parietal, temporal, Occipital.	Yes	Yes	20
7	Spontaneous	~	Visual, 120	Yes	Bilateral	Visual: Flicking	~	Right side: Frontal Left side: Frontal.	~	Yes	-60
	CGRP infusion	30	Visual, 330	Yes	Bilateral	Visual: Flickering	10	Left side: Frontal and temporal.	Yes	Yes	20
8	Spontaneous	~	Visual, 10 Sensory, 30	Yes	Visual: Left side Sensory: Left or right side	Visual: Scotoma, flickering Sensory: Numbness in hand, foot, leg	~	Left side: Periorbital.	~	Yes	-45
	CGRP infusion	10	Visual, 40 Sensory, 15	Visual: Yes Sensory: No	Visual: Left side Sensory: bilateral, hands	Visual: Flickering Sensory: Tingling, numbness	20	Left side: Frontal, occipital.	Yes	Yes	-10
9	Spontaneous	~	Visual, 60 Sensory, 1440	Yes	Visual: Right side Sensory: Right side	Visual: scotoma, zigzag lines, firework Sensory: Numbness in face, arm, hand	~	Right side: Frontal, periorbital, temporal, occipital.	~	Yes	-60
	CGRP infusion	20	Visual, 30 Sensory, 520	Yes	Visual: Right side. Sensory: Right side, face, tongue, arm, hand	Visual: Flickering blue light Sensory: Tingling	10	Rigt side: Frontal.	Yes	Yes	10
10	Spontaneous	~	Visual, 60 Sensory, 150	Yes	Visual: Bilateral Sensory: Right side face, hand	Visual: Scotoma, zigzag lines, shining light and black dots Sensory: Tingling	~	Right side: Periorbital, occipital.	~	Yes	-60
	CGRP infusion	20	Visual, 10 min Sensory, 40 min	Yes	Visual: Bilateral Sensory: Right side face, hand	Visual: Shining light and black dots Sensory: Tingling, numbness	10	Right side: Periorbital, temporal, occipital.	Yes	Yes	10
11	Spontaneous	~	Visual, 20 Sensory, 30	Yes	Visual: Bilateral Sensory: Left side, face, hand, arm, foot, leg	Visual: Flickering light, zigzag lines Sensory: Tingling, numbness	~	Right side: Frontal, periorbital, parietal, temporal.	~	Yes	-15
	CGRP infusion	30	Sensory, 45	Yes	Left side, fingers, arm, face	Tingling	10	Right side: Frontal, temporal.	Yes	Yes	20
12	Spontaneous	~	Visual, 60 Sensory, 30	Yes	Visual: Bilateral Sensory: Right side face, hand, arm	Visual: Scotoma, flickering light, zigzag lines Sensory: Numbness	~	Right side: Frontal, periorbital, temporal. Left side: Frontal, periorbital, temporal.	~	Yes	-60
	CGRP infusion	20	Visual, 50	Yes	Bilateral	Flickering	10	Right side: Frontal, temporal. Left side: Frontal, temporal.	Yes	Yes	10
13	Spontaneous	~	Visual, 210	Yes	Bilateral	Visual: Scotoma, flickering light	~	Right side: Frontal, periorbital, temporal.	~	Yes	-45
	CGRP infusion	30	Visual, 40	No	Left side	Flickering	10	Right side: Frontal, parital, occipital.	Yes	Yes	20

*CGRP *Calcitonin gene-related peptide

~: Not applicable

**Fig. 1:**
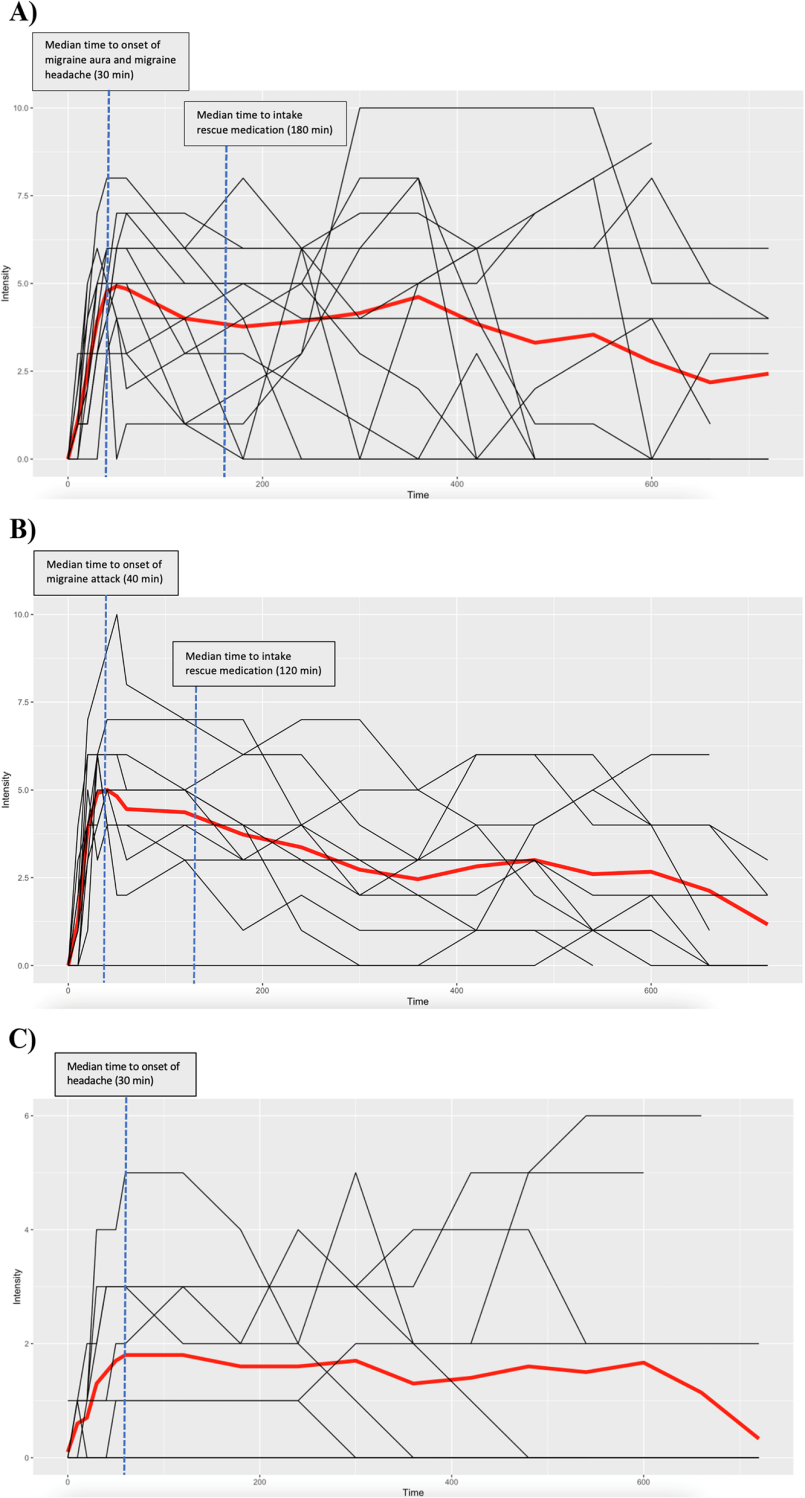
** A **Headache intensity from 0 min to 12 h in participants who reported migraine aura and migraine headache following calcitonin gene-related peptide infusion, The abscissa refers to the time (min). Median (thick, red line) and individual (thin, black lines) headache intensity on NRS. The median time to onset of migraine aura and migraine headache was 30 min (IQR aura, 20 to 30; IQR headache, 20 to 53). The median time to intake rescue medication was 180 min (IQR, 120 to 300). **B** Headache intensity from 0 min to 12 h in participants who only reported migraine headache following calcitonin gene-related peptide infusion. The abscissa refers to the time (min). Median (thick, red line) and individual (thin, black lines) headache intensity on NRS. The median time to onset of migraine headache was 40 min (IQR, 20 to 120). The median time to intake rescue medication was 120 min (IQR, 60 to 330). **C** Headache intensity from 0 min to 12 h in participants who did not report migraine aura or migraine headache following calcitonin gene-related peptide infusion. The abscissa refers to the time (min). Median (thick, red line) and individual (thin, black lines) headache intensity an NRS. The median time to onset of headache was 10 min (IQR, 10 to 20). None of these participants took rescue medication

 All 13 participants reported that the CGRP-induced migraine aura closely resembled their typical spontaneous migraine attacks with aura. Among these participants, 10 reported using current preventive medication for migraine. The median time to onset of CGRP-induced migraine aura was 30 min (IQR, 20 to 30) after the start of the infusion (Fig. 1A-C). Twelve of the 13 participants developed migraine aura and migraine headache, while 1 participant developed migraine aura and a headache of severe intensity, but the headache characteristics did not meet the criteria for migraine headache outlined in ICHD-3 (see the Supplemental Appendix Table 3). Regarding migraine headache, the median time to onset was 30 min (IQR, 20 to 53). Moreover, participants who experienced CGRP-induced migraine aura had a higher median number of aura attacks in the past year (60; IQR, 36 to 96), compared with those who did not develop CGRP-induced migraine aura (9; IQR, 5 to 60) (*P* = 0.03) (Table 1).

### Migraine Headache

During the 12-hour observational period following CGRP infusion, 24 (71%) of 34 participants reported experiencing migraine headache (see the Supplemental Appendix Table 3). As mentioned previously, 12 of these 24 participants also developed migraine aura along with migraine headache. The median time to onset of migraine headache was 40 min (IQR, 20 to 120) after the start of the infusion, and the median peak intensity of the headache was 6 (IQR, 6 to 7). Among the 24 participants who experienced CGRP-induced migraine headache, 13 (54%) reported currently using preventive medication for migraine.

### Vital signs, adverse events, and rescue medication

During the 60-minute in-hospital phase, the mean percentagewise increase in heart rate was 38% ± 22%, and the mean percentagewise decrease in blood pressure was 12% ± 12% (see the Supplemental Appendix Fig. 1). Furthermore, post-infusion start, the most common adverse events reported were flushing (*n* = 33 [97%]), warm sensations (*n* = 32 [94%]), heart palpitations (*n* = 21 [62%]), neck pain (*n* = 20 [59%]), and unusual tiredness (*n* = 8 [24%]). Moreover, 17 (50%) of 34 required rescue medication to treat their headache.

## Discussion

Our study revealed that 13 (38%) of 34 participants developed migraine aura following intravenous infusion of CGRP. Importantly, all induced migraine aura attacks were reported to closely resemble the participants’ usual spontaneous migraine attacks with aura. Another notable observation was that those who experienced migraine aura after CGRP infusion had a higher frequency of migraine aura in the past year, compared with those who did not. These findings provide preliminary evidence indicating that further exploration of therapeutics targeting CGRP signalling could potentially be beneficial in preventing migraine attacks with aura.

### CGRP and aura

An intriguing finding from a previous experimental study is that intravenous infusion of CGRP induced migraine aura in 4 (29%) of 14 individuals exclusively diagnosed with migraine with aura [10]. This rate is less than the 38% induction rate in our study. In contrast to the prior study, our participants had a much higher frequency of migraine attacks with aura in the past year, and most of them also experienced migraine attacks without aura. The relationship between the frequency of aura and the coexistence of migraine with and without aura and the susceptibility to develop CGRP-induced migraine aura remains unclear. However, our findings suggest that a higher frequency of aura may increase the likelihood of developing CGRP-induced migraine aura. Table 1 shows no variations between individuals who responded to CGRP infusion and those who did not. Due to the limited size of participants in each grope, no statistical analyses were performed.

**Fig. 2:**
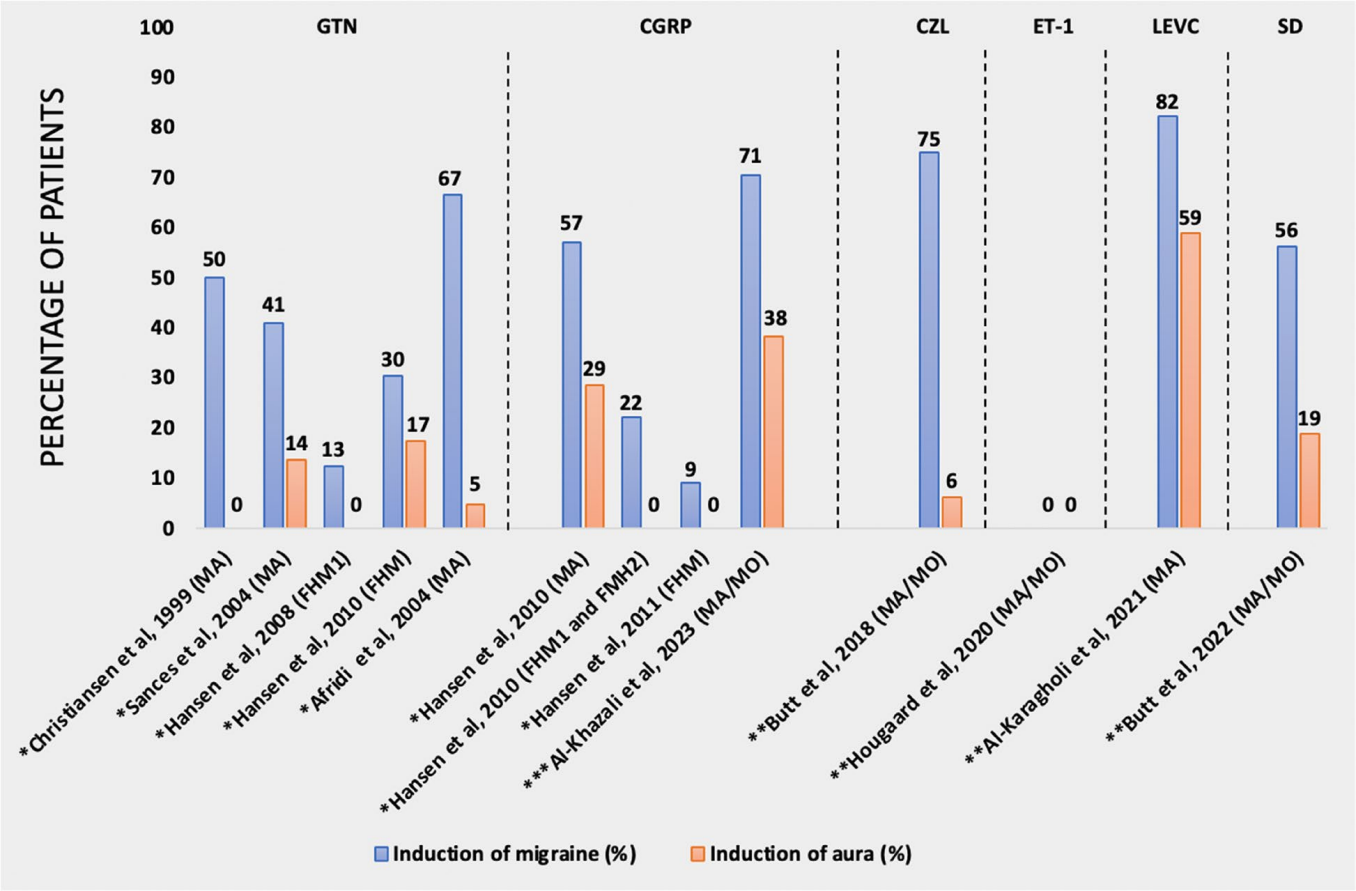
Human provocation studies investigating migraine aura. *Healthy controls as comparison; **placebo; ***no comparison. GTN, glyceryl trinitrate; CGRP, calcitonin gene-related peptide; CZL, Cilostazol; ET-1, Endothelin-1; LEVC, levcromakalim; SD, Sildenafil. MA, migraine aura exclusively; MA/MO, migraine aura, but not exclusively migraine aura; FHM, familial hemiplegic migraine (unknown mutations); FHM1, familial hemiplegic migraine with known mutations (R583Q and C1369Y mutations); FHM2, familial hemiplegic migraine with known mutations (R202Q and R763C mutations)

 When considering the interpretation of our results, it is essential to recognize the potential influence of nocebo effects. This may arise from various factors, including individual psychosocial variables (such as anxiety and stress), the open-label design of our study, and the occurrence of spontaneous attacks. However, in 4 randomized, placebo-controlled trials, migraine aura was only reported in 1 (2%) of 61 participants who received placebo infusion (isotonic saline) (Fig. 2; Table 3) [15–18]. This suggests that the aura induction rate we observed in our study is less likely to be a result of nocebo effects. Furthermore, it is improbable that the open-label design had a significant impact on the nocebo effect for migraine aura. Across 3 open-label studies involving 55 participants with migraine aura who received glyceryl trinitrate (GTN), only 4 (7%) participants developed migraine aura [19–21]. Pooling our previous study [10] and the current one brings the total number of participants who received CGRP to 48, yielding an aura induction rate of 35%, which is higher than that observed after GTN administration. This suggests a response higher than what would be typically expected from nocebo effects among patients with migraine aura.

**Table 3:** Human provocation studies investigating migraine aura

Author, Year	Substance	Total number of participants	Migraine induction rate (N)	Aura induction rate (N)	Frequency of aura attacks (range, pr year)
^a^Christiansen et al., 1999 (MA) [21]	GTN	12	6	0	7 to 50
^a^Sances et al., 2004 (MA) [20]	GTN	22	9	3	NA
^a^Afridi et al., 2004 (MA) [19]	GTN	21	14	1	NA
^a^Hansen et al., 2008 (FHM1) [22]	GTN	8	1	0	< 1 to 10
^a^Hansen et al., 2010 (FHM) [23]	GTN	23	7	4	NA
^a^Hansen et al., 2010 (MA) [10]	CGRP	14	8	4	2 to 24
^a^Hansen et al., 2008 (FHM1 and FMH2) [24]	CGRP	9	2	0	< 1 to 10
^a^Hansen et al., 2011 (FHM) [25]	CGRP	11	1	0	2 to 15
^c^Al-Khazali et al., 2023 (MA/MO)	CGRP	34	24	13	1 to 179
^b^Butt et al., 2018 (MA/MO) [15]	Cilostazol	16	12	1	2 to 12
^b^Hougaard et al., 2020 (MA/MO) [17]	Endothelin-1	12	0	0	12 to 48
^b^Al-Karagholi et al., 2021 (MA) [18]	Levcromakalim	17	14	10	6 to 50
^b^Butt et al., 2022 (MA/MO) [16]	Sildenafil	16	9	3	2 to 12

*GTN *glyceryl trinitrate,* CGRP *calcitonin gene-related peptide, *MA *migraine aura exclusively,* MA/MO m*igraine aura, but not exclusively migraine aura* FHM * familial hemiplegic migraine (unknown mutations)* FHM1 *familial hemiplegic migraine with known mutations (R583Q and C1369Y mutations),* FHM2 *familial hemiplegic migraine with known mutations (R202Q and R763C mutations)

^a^Healthy controls as comparison

^b^placebo

^c^no comparison

It is important to ascertain that the reported migraine auras following CGRP administration were not spontaneous. Given that the median number of migraine aura days in the previous year was 25, the probability of this seems low. With an induction rate of 38%, one would anticipate a median of 139 migraine aura days per year. Another key argument against the occurrence of spontaneous migraine aura is that the median time to onset of CGRP-induced migraine aura was 30 min. Furthermore, among the 13 participants who experienced migraine aura, 10 of them had the aura onset within 1 h of starting the infusion. Given the rapid aura onset following CGRP infusion, it is challenging to argue that spontaneous migraine aura coincidentally occurred during this period. Nevertheless, future placebo-controlled studies are warranted to further confirm migraine aura inducing effects of CGRP.

Our study uncovers a new link between the frequency of aura episodes and susceptibility to CGRP-triggered migraine aura, shedding new light on this complex relationship. Moreover, our data rule out the possibility that the migraine aura induced by CGRP infusion is simply a nocebo effect, removing a significant confounding factor and strengthening the credibility of our conclusions. Finally, we show that the observed migraine aura following CGRP administration is not likely to be spontaneous, given its rapid onset. This finding sets our work apart from previous research and adds to our understanding of CGRP-induced migraine aura, providing a more detailed view that could guide future treatment approaches.

### Aura-headache linkage

In the present trial, intravenous infusion of CGRP induced immediate onset of headache in all participants. The onset of migraine aura and migraine headache followed soon after, with 4 participants reporting migraine headache before aura, 3 simultaneously with aura, and 5 after aura onset. Interestingly, 10 of 13 participants who experienced CGRP-induced migraine aura reported that headache sometimes preceded or coincided with the onset of their usual spontaneous migraine attacks with aura. This accords well with an observational study reporting that half of patients with migraine with aura experience migraine headache at the time of aura onset or within 15 min [26].

The linkage between migraine aura and migraine headache remains a topic of ongoing research, marked by the complexities in translating CSD features from animals to clinical features of migraine aura, as well as the inherent difficulties in measuring CSD in humans. Despite these challenges, two concepts have been proposed to explain the aura-headache relationship [27–29]. The first concept is known as the sequential concept, which suggests that CSD, manifested as aura, promotes the dilation of intracranial blood vessels and neurogenic inflammation, leading to the development of migraine headache [29]. The second concept is the parallel concept, which proposes that migraine aura and migraine headache are separate events occurring in close temporal relation and in response to a shared initiating factor [27, 28]. Building upon these existing theories, our study provides new insights having identified CGRP as a trigger of not only migraine headache but also migraine aura.

### Neurobiological underpinnings of CGRP-induced migraine aura

Within the CNS, CGRP and its receptors are found in structures that are central to headache pathophysiology, including the trigeminocervical complex (TCC) and the thalamus [30]. CGRP’s role extends beyond vascular effects, as it modulates nociceptive transmission within the second-order neurons of the TCC in the trigeminovascular pathway [31, 32]. Experimental evidence has shown that introducing CGRP into the periaqueductal gray area can enhance TCC nociceptive transmission [33], while CGRP antagonism has the opposite effect, inhibiting this transmission [32]. In rodent models, the cortical application of potassium chloride has been found to induce CSD and CGRP release [34]. Interestingly, CGRP antagonism has been shown to prevent the activation and sensitization of central trigeminovascular neurons activated by CSD [34]. This finding adds a layer of complexity to our understanding of CGRP’s role in migraine. It is also important to acknowledge that while both fremanezumab and atogepant have failed to completely prevent CSD, they have demonstrated the ability to slow down the propagation of CSD and reduce the duration of cortical recovery following the event [35–37]. This understanding of CGRP’s dual role in vascular and non-vascular processes provides important knowledge of migraine pathophysiology and may shape the direction of upcoming therapeutic approaches. In summary, the understanding of CGRP’s dual role in vascular and non-vascular processes provides important knowledge of migraine pathophysiology and may shape the direction of upcoming therapeutic approaches.

Our findings raise an important question regarding the mechanism by which intravenously administered CGRP induces migraine aura, specifically in relation to the CSD. The molecular size of CGRP suggests a limited capability to penetrate the blood-brain barrier, a concept further supported by the absence of middle cerebral artery dilation observed in humans following intravenous infusion [38, 39].

To explain the present results, we therefore propose that intravenously administered CGRP reaches the meningeal arteries and binds to its G protein-coupled receptors on the vascular smooth muscle cells [2, 40, 41]. This binding activates cAMP-dependent pathways, leading to the opening of specific potassium channels, such as ATP-sensitive potassium channels (a recently discovered trigger of migraine aura) [18, 40]. The efflux of potassium then promotes dilation of the meningeal arteries [18, 40, 42]. The combination of increased extracellular potassium and vasodilation is hypothesized to provide chemical and mechanical stimuli, respectively, which activates and sensitizes perivascular primary afferents [2, 41]. The latter transmits nociceptive information to certain cortical and subcortical areas (e.g., somatosensory cortex, visual cortex) through second-order neurons in the brain stem and third-order neurons in the thalamus [2, 41]. Ultimately, the somatosensory cortex and other cortical/subcortical areas are responsible for generating perceptions of cephalic pain, including migraine headache. This proposed sequence of events provides a potential explanation for CGRP-induced migraine headache but might also explain the genesis of CGRP-induced migraine aura, as detailed below.

We hypothesize that the excitatory stimuli reaching areas such as the visual cortex might reach the threshold for CSD initiation in susceptible individuals. Preclinical data does indeed suggest that axonal projections to the visual cortex release the excitatory neurotransmitter glutamate, which lower the threshold for CSD initiation [43–45]. Also, functional magnetic resonance imaging studies have demonstrated that nociceptive trigeminal stimulation might activate the visual cortex [38, 46, 47]. This is congruent with preclinical data showing that trigeminal and visual input converge at the level of third-order thalamic neurons, which then project to the visual cortex [43, 48]. Interestingly, visual stimulation with a checkerboard has been shown to elicit migraine aura in people with migraine with aura and this was associated with blood oxygenation level-dependent (BOLD) changes resembling CSD [49–52]. Taken together, it seems reasonable to assert that CGRP-induced activation of ascending cephalic pain pathways might provide sufficient excitatory stimuli to areas such as the visual cortex which, in turn, results in CSD initiation. Further translational research is warranted to explore the relationship between migraine aura and headache, including what role CGRP plays in this context.

On another note, it is interesting to consider the data regarding the prevention of migraine aura with the use of monoclonal antibodies targeting CGRP signalling [7, 53]. Some evidence suggests that these medications are also effective in people with history of aura, providing additional support for our assertion that CGRP can induce aura without crossing the blood-brain barrier. This is because monoclonal antibodies are much larger molecules than CGRP and have very limited ability to penetrate brain barriers. However, caution is warranted as the efficacy data of these drugs in preventing migraine aura is currently limited by small sample sizes and inadequate methods for evaluating aura [7, 53].

### Limitations

We recognize several constraints in our study. A significant portion of participants reported ongoing use of preventative migraine medications, which may have influenced their responsiveness to CGRP infusion. Most participants came from a tertiary referral center, which might limit the applicability of our results to a broader population of individuals with migraine aura. We also omitted an a priori sample size calculation, emphasizing that our study’s primary aim was exploratory. The absence of a placebo arm could have influenced participants’ expectations and responses regarding CGRP-induced migraine aura attacks.

## Conclusions

Our study presents novel evidence that intravenous CGRP infusion can induce migraine aura in people with migraine with aura. This indicates that CGRP can trigger CSD, possibly by initiating interactive processes between ascending cephalic pain pathways and CSD-susceptible areas, such as the visual cortex. Moreover, our findings highlight the potential of CGRP-targeted drugs in preventing both migraine headache and aura. Further research is required to unravel the underlying mechanisms of CGRP-induced migraine aura and headache.

### Supplementary Information

**Additional File 1:** **Supplementary Material. Figure 1.** MAP and HR after infusion of calcitonin gene-related peptide. **Table 1.** Inclusion and exclusion criteria. **Table 2A. **Criteria B and C for migraine aura according to ICHD-3. **Table 2B.** Criteria C and D for migraine without aura according to ICHD-3. **Table 3. **Clinical characteristics of headache and associated symptoms in participants with migraine after calcitonin gene-related peptide provocation.

## Data Availability

Upon reasonable request, the corresponding author will provide the necessary data and materials to interested researchers for the purpose of academic scrutiny, reproducibility, and further scientific investigation.

## Abbreviations

CGRP: Calcitonin gene-related peptide
CSD: Cortical spreading depolarization
REFORM: Registry for Migraine
ICHD-3: International Classification of Headache Disorders, 3rd edition
IQR: Interquartile range
FHM: Familial hemiplegic migraine
TCC: Trigeminocervical complex
BOLD: Blood oxygenation level-dependent
