# Supplementary material for: Calcitonin gene-related peptide causes migraine aura
Source: J Headache Pain. 2023 Sep 7;24(1):124. doi: 10.1186/s10194-023-01656-4 (PMC10483878; doi:10.1186/s10194-023-01656-4)
Supplement: Supplementary file 1 — Additional File 1: Supplementary Material. Figure 1. MAP and HR after infusion of calcitonin gene-related peptide. Table 1. Inclusion and exclusion criteria. Table 2A. Criteria B and C for migraine aura according to ICHD-3. Table 2B. Criteria C and D for migraine without aura according to ICHD-3. Table 3. Clinical characteristics of headache and associated symptoms in participants with migraine after calcitonin gene-related peptide provocation. [file 10194_2023_1656_MOESM1_ESM.docx]

**Supplementary Materials**

**Figures**

**Figure 1 – MAP and HR after infusion of calcitonin gene-related peptide.**


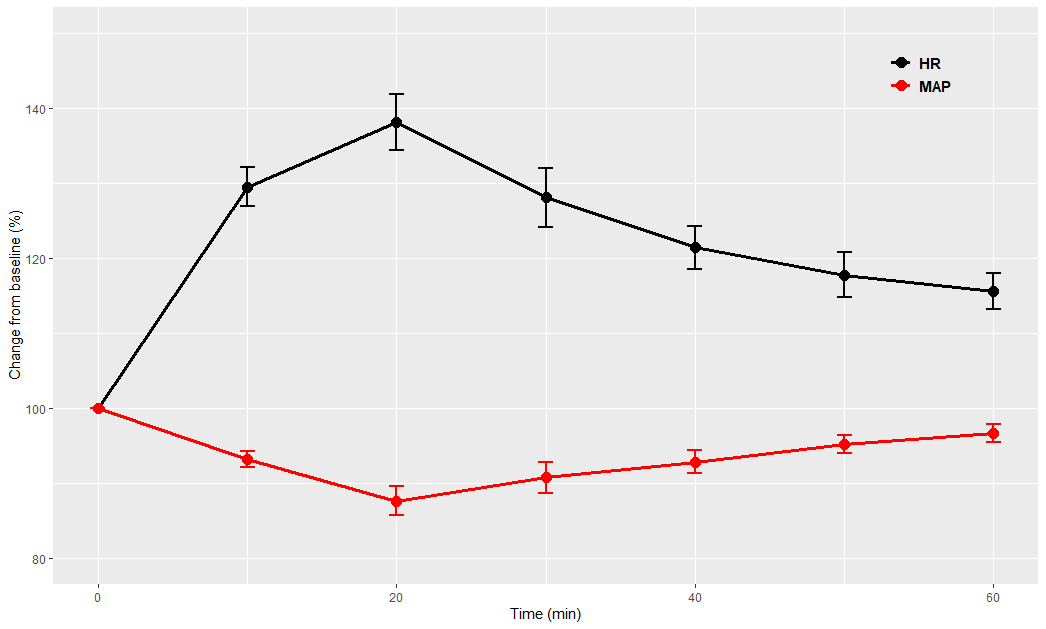


The black line represents the mean heart rate, while the red line represents the mean arterial blood pressure.

**Tables**

**Table 1 – Inclusion and exclusion criteria.**

| Inclusion criteria |
| --- |
| - Age ≥ 18 years of age upon entry into screening. - History of migraine with or without aura for ≥ 12 months according to the International Classification of Headache Disorders 3rd Edition (ICHD-3) criteria. - ≥ 4 headache days that meet criteria as migraine days per month on average across the 3 months before screening. - Subject has provided informed consent prior to initiation of any study-specific activities/procedures. |
| Exclusion criteria |
| - > 50 years of age at migraine onset. - History of cluster headache or hemiplegic migraine. - Inability to differentiate migraine from other headaches. - The subject is at risk of self-harm or harm to others as evidenced by past suicidal behavior. - History or evidence of any other clinically significant disorder, condition or disease that, in the opinion of the site investigator, would pose a risk to subject safety or interfere with study evaluation, procedures or completion. - Female subjects of childbearing potential with a positive pregnancy test during any study visit. - Female subject is pregnant or breastfeeding or planning to become pregnant during the study. - Evidence of current pregnancy or breastfeeding. - Female subject of childbearing potential unwilling to use 1 acceptable method of effective contraception. - Hypertension on the experimental day defined as systolic blood pressure ≥ 150mmHg or diastolic blood pressure ≥ 100 mmHg. - Hypotension on the experimental day defined as systolic blood pressure < 90mmHg or diastolic blood pressure < 50 mmHg. - Any headache (including migraine) within 24 hours prior to the start of the CGRP-infusion. - Intake of any analgesics or migraine-specific medications within 24 hours prior to the start if the CGRP-infusion. - Previously treatment with erenumab. - Treatment with another anti-CGRP monoclonal antibody for three months prior to screening. - Concomitant preventive medication apart from anti-CGRP antibodies was allowed, but only if dosage was stable for two months prior to the screening. |

**Table 2A – Criteria B and C for migraine aura according to ICHD-3.**

|  |
| --- |
| B. One or more of the following fully reversible aura symptoms:   - Visual - Sensory - Speech and/or language - Motor - Brainstem - Retinal |
| C. At least three of the following six characteristics:   - At least one aura symptom spreads gradually over ≥5 minutes. - Two or more aura symptoms occur in succession. - Each individual aura symptom lasts 5-60 minutes. - At least one aura symptom is unilateral. - At least one aura symptom is positive. - The aura is accompanied, or followed within 60 minutes, by headache. |

**Table 2B – Criteria C and D for migraine without aura according to ICHD-3.**

|  |
| --- |
| C. Headache has at least two of the following four characteristics:   - Unilateral location. - Pulsating quality. - Moderate or severe pain intensity. - Aggravation by or causing avoidance of routine physical activity (eg, walking or climbing stairs). |
| D. During headache at least one of the following:   - Nausea and/or vomiting. - Photophobia and phonophobia |

**Table 3 – Clinical characteristics of headache and associated symptoms in participants with migraine after calcitonin gene-related peptide provocation.**

|  |  |  |  | Headache characteristics | | | | Associated symptoms | | |  |  |  |  |  |
| --- | --- | --- | --- | --- | --- | --- | --- | --- | --- | --- | --- | --- | --- | --- | --- |
|  | Participants no. |  | **Peak headache, min** | **Localizatio~** | **Peak intensity** | **Quality (0=pressing, 1=pulsating)** | **Aggravated by cough or movement** | **Nausea** | **Photophobia** | **Phonophobia** | **Mimics usual migraine** | **Onset of migraine attack, min** | **Onset of migraine attack Intensity** | **Treatment (time, min) / efficacy^b,c^** | **Current**  **use of preventive medication** |
| CGRP induced migraine aura and headache | 1 | Spontaneous | ~ | Unilateral | 8 | Pulsating | Yes | Yes | Yes | Yes | ~ | ~ | ~ | ~ | ~ |
|  |  | CGRP infusion | 300 | Bilateral | 10 | Pulsating | Yes | Yes | Yes | Yes | Yes | 180 | 1 | Treo* (420)  Eletriptan (420)  / No | ~ |
|  | 2 | Spontaneous | ~ | Right | 8 | Pressing | Yes | Yes | Yes | Yes | ~ | ~ | ~ | ~ | Amitriptylin |
|  |  | CGRP infusion | 600 | Bilateral | 9 | Pressing | Yes | Yes | Yes | Yes | Yes | 20 | 5 | No | Amitriptylin |
|  | 3 | Spontaneous | ~ | Unilateral | 10 | Pulsating | Yes | Yes | Yes | Yes | ~ | ~ | ~ | ~ | Lisinopril |
|  |  | CGRP infusion | 50 | Bilateral | 7 | Pulsating | Yes | Yes | Yes | Yes | Yes | 10 | 1 | No | Lisinopril |
|  | 4 | Spontaneous | ~ | Unilateral | 8 | Pulsating | Yes | Yes | Yes | Yes | ~ | ~ | ~ | ~ | Botox |
|  |  | CGRP infusion | 360 | Right | 8 | Pulsating | Yes | Yes | Yes | Yes | Yes | 20 | 1 | Zolmitriptan (300)  / No | Botox |
|  | 5 | Spontaneous | ~ | Unilateral | 8 | Pulsating | Yes | Yes | Yes | Yes | ~ | ~ | ~ | ~ | Amitriptylin |
|  |  | CGRP infusion | 40 | Bilateral | 6 | Pulsating | No | Yes | Yes | Yes | Yes | 40 | 6 | Zolmitriptan (240)  / Yes | Amitriptylin |
|  | 6 | Spontaneous | ~ | Unilateral | 6 | Pulsating | Yes | Yes | Yes | Yes | ~ | ~ | ~ | ~ | Candesartan |
|  |  | CGRP infusion | 30 | Bilateral | 6 | Pulsating | Yes | Yes | Yes | Yes | Yes | 10 | 2 | Paracetamol (40)  / No | Candesartan |
|  | 7 | Spontaneous | ~ | Unilateral | 8 | Pressing | Yes | Yes | Yes | Yes | ~ | ~ | ~ | ~ | ~ |
|  |  | CGRP infusion | 300 | Left | 8 | Pressing | Yes | Yes | Yes | Yes | Yes | 240 | 5 | Eleptriptan (300)  / Yes | ~ |
|  | 8* | Spontaneous | ~ | Unilateral | 9 | Pulsating | Yes | Yes | Yes | No | ~ | ~ | ~ | ~ | Candesartan |
|  |  | CGRP infusion | 600 | Left | 8 | Pulsating | Yes | No | Yes | No | No | No | No | No | Candesartan |
|  | 9 | Spontaneous | ~ | Right | 10 | Pulsating | Yes | Yes | Yes | Yes | ~ | ~ | ~ | ~ | Candesartan |
|  |  | CGRP infusion | 50 | Bilateral | 6 | Pulsating | Yes | No | Yes | Yes | Yes | 50 | 6 | Rizatriptan (120)  / No | Candesartan |
|  | 10 | Spontaneous | ~ | Right | 8 | Pressing | Yes | Yes | Yes | Yes | ~ | ~ | ~ | ~ | Riboflavin, Magnesium |
|  |  | CGRP infusion | 240 | Bilateral | 6 | Pressing | Yes | Yes | Yes | Yes | Yes | 20 | 3 | Treo (120)  / No | Riboflavin, Magnesium |
|  | 11 | Spontaneous | ~ | Bilateral | 7 | Pulsating | Yes | Yes | Yes | Yes | ~ | ~ | ~ | ~ | Lamotrigin |
|  |  | CGRP infusion | 60 | Bilateral | 7 | Pulsating | Yes | Yes | Yes | Yes | Yes | 20 | 4 | Rizatriptan (480)  / Yes | Lamotrigin |
|  | 12 | Spontaneous | ~ | Unilateral | 9 | Pulsating | Yes | Yes | Yes | Yes | ~ | ~ | ~ | ~ | Amitriptylin |
|  |  | CGRP infusion | 40 | Bilateral | 8 | Pulsating | Yes | Yes | Yes | Yes | Yes | 40 | 8 | Rizatriptan (120)  / Yes | Amitriptylin |
|  | 13 | Spontaneous | ~ | Right | 8 | Pulsating | Yes | Yes | Yes | Yes | ~ | ~ | ~ | ~ | ~ |
|  |  | CGRP infusion | 50 | Bilateral | 5 | Pulsating | No | No | No | No | Yes | 60 | 5 | Rizatriptan (60)  / Yes | ~ |
| CGRP induced migraine headache | 1 | Spontaneous | ~ | Unilateral | 7 | Pulsating | Yes | No | Yes | Yes | ~ | ~ | ~ | ~ | ~ |
|  |  | CGRP infusion | 40 | Bilateral | 5 | Pressing | No | No | No | No | Yes | 120 | 3 | Treo (120)  Sumatriptan (120)  / No | ~ |
|  | 2 | Spontaneous | ~ | Unilateral | 10 | Pulsating | Yes | Yes | Yes | Yes | ~ | ~ | ~ | ~ | ~ |
|  |  | CGRP infusion | 600 | Right | 2 | Pulsating | Yes | Yes | Yes | Yes | Yes | 540 | 1 | Ibuprofen (540)  / Yes | ~ |
|  | 3 | Spontaneous | ~ | Bilateral | 8 | Pulsating | Yes | Yes | Yes | Yes | ~ | ~ | ~ | ~ | Amitriptylin, Topiramat |
|  |  | CGRP infusion | 20 | Bilateral | 5 | Pulsating | Yes | Yes | Yes | Yes | Yes | 120 | 3 | No | Amitriptylin, Topiramat |
|  | 4 | Spontaneous | ~ | Unilateral | 6 | Pulsating | Yes | Yes | Yes | Yes | ~ | ~ | ~ | ~ | ~ |
|  |  | CGRP infusion | 30 | Bilateral | 5 | Pulsating | Yes | Yes | Yes | Yes | Yes | 20 | 4 | Treo (120)  / No | ~ |
|  | 5 | Spontaneous | ~ | Unilateral | 5 | Pulsating | Yes | Yes | Yes | Yes | ~ | ~ | ~ | ~ | ~ |
|  |  | CGRP infusion | 30 | Bilateral | 6 | Pulsating | Yes | Yes | Yes | No | Yes | 20 | 4 | No | ~ |
|  | 6 | Spontaneous | ~ | Right | 10 | Pulsating | Yes | Yes | Yes | Yes | ~ | ~ | ~ | ~ | ~ |
|  |  | CGRP infusion | 30 | Bilateral | 6 | Pulsating | Yes | Yes | No | No | No | 20 | 1 | No | ~ |
|  | **7** | Spontaneous | ~ | Unilateral | 9 | Pulsating | Yes | Yes | Yes | Yes | ~ | ~ | ~ | ~ | Candesartan, Magnesium |
|  |  | CGRP infusion | 180 | Bilateral | 6 | Pulsating | Yes | Yes | Yes | Yes | No | 120 | 5 | No | Candesartan, Magnesium |
|  | 8 | Spontaneous | ~ | Right | 8 | Pressing | Yes | Yes | Yes | Yes | ~ | ~ | ~ | ~ | Amitriptylin, Botox |
|  |  | CGRP infusion | 20 | Bilateral | 6 | Pressing | Yes | Yes | Yes | Yes | Yes | 120 | 4 | Domperiodon (420)  Parcetamol (540)  Ibuprofen (540)  / Yes | Amitriptylin, Botox |
|  | 9 | Spontaneous | ~ | Right | 6 | Pulsating | Yes | Yes | Yes | Yes | ~ | ~ | ~ | ~ | ~ |
|  |  | CGRP infusion | 40 | Bilateral | 7 | Pulsating | Yes | Yes | No | No | Yes | 40 | 7 | Treo (120)  / No | ~ |
|  | 10 | Spontaneous | ~ | Bilateral | 9 | Pulsating | Yes | Yes | Yes | Yes | ~ | ~ | ~ | ~ | ~ |
|  |  | CGRP infusion | 50 | Bilateral | 10 | Pulsating | Yes | Yes | Yes | Yes | Yes | 20 | 7 | Domperiodon (60)  / No | ~ |
|  | 11 | Spontaneous | ~ | Bilateral | 8 | Pulsating | Yes | Yes | Yes | Yes | ~ | ~ | ~ | ~ | ~ |
|  |  | CGRP infusion | 20 | Bilateral | 6 | Pulsating | Yes | Yes | Yes | Yes | Yes | 20 | 6 | Ibuprofen (50)  Sumatriptan (50)  / No | ~ |
| CGRP did not induce migraine with or without aura | 1 | Spontaneous | ~ | Bilateral | 7 | Pulsating | Yes | Yes | No | No | ~ | ~ | ~ | ~ | ~ |
|  |  | CGRP infusion | 10 | Bilateral | 1 | Pressing | No | No | No | No | No | No | No | No | ~ |
|  | 2 | Spontaneous | ~ | Bilateral | 8 | Pressing | No | Yes | No | No | ~ | ~ | ~ | ~ | ~ |
|  |  | CGRP infusion | 540 | Bilateral | 6 | Pressing | Yes | No | No | Yes | No | No | No | No | ~ |
|  | 3 | Spontaneous | ~ | Unilateral | 6 | Pressing | Yes | Yes | Yes | Yes | ~ | ~ | ~ | ~ | Botox |
|  |  | CGRP infusion | 240 | Bilateral | 4 | Pressing | No | Yes | Yes | No | No | No | No | No | Botox |
|  | 4 | Spontaneous | ~ | Unilateral | 5 | Pressing | No | Yes | Yes | Yes | ~ | ~ | ~ | ~ | ~ |
|  |  | CGRP infusion | No | No | 0 | No | No | No | No | No | No | No | No | No | ~ |
|  | 5 | Spontaneous | ~ | Left | 9 | Pressing | Yes | Yes | Yes | No | ~ | ~ | ~ | ~ | Candesartan |
|  |  | CGRP infusion | No | No | 0 | No | No | No | No | No | No | No | No | No | Candesartan |
|  | 6 | Spontaneous | ~ | Unilateral | 9 | Pulsating | Yes | Yes | Yes |  | ~ | ~ | ~ | ~ | Candesartan |
|  |  | CGRP infusion | 360 | Bilateral | 4 | Pressing | Yes | No | Yes | No | No | No | No | No | Candesartan |
|  | 7 | Spontaneous | ~ | Bilateral | 8 | Pressing | Yes | Yes | Yes | Yes | ~ | ~ | ~ | ~ | ~ |
|  |  | CGRP infusion | 300 | Bilateral | 2 | Pressing | Yes | No | Yes | No | No | No | No | No | ~ |
|  | 8 | Spontaneous | ~ | Right | 8 | Pulsating | Yes | Yes | Yes | Yes | ~ | ~ | ~ | ~ | ~ |
|  |  | CGRP infusion | 420 | Right | 5 | Pulsating | Yes | Yes | Yes | Yes | Yes | No | No | No | ~ |
|  | 9 | Spontaneous | ~ | Right | 8 | Pressing | Yes | Yes | Yes | Yes | ~ | ~ | ~ | ~ | ~ |
|  |  | CGRP infusion | 40 | Bilateral | 3 | Pressing | Yes | No | No | No | Yes | No | No | No | ~ |
|  | 10 | Spontaneous | ~ | Bilateral | 8 | Pressing | Yes | Yes | Yes |  | ~ | ~ | ~ | ~ | Lamotrigin |
|  |  | CGRP infusion | 50 | Bilateral | 1 | Pressing | Yes | No | Yes | No | No | No | No | No | Lamotrigin |

CGRP: calcitonin gene-related peptide

a: Unilateral = Headache can occur on right or left side

b: Yes = Pain freedom or pain relief (≥ 50% decrease of intensity) within 2h.

c: Treo = Combination of Acetylsalicylic acid (500 mg) and Caffein (50 mg)

~: Not applicable

* Subject 8 developed headache and aura following CGRP infusion, but the headache characteristics did not fulfill the migraine criteria outlined in ICHD-3.
